# Evaluation of deliverable dose-mimicking automated volumetric arc radiation therapy planning for stage III non-small cell lung cancer patients: comparison with a commercial DVH-predicted automated planning system

**DOI:** 10.1093/jrr/rrag001

**Published:** 2026-02-05

**Authors:** Takeru Nakajima, Noriyuki Kadoya, Ryota Tozuka, Masaki Kondo, Shohei Tanaka, Kazuhiro Arai, Yoshiyuki Katsuta, Taichi Hoshino, Takaya Yamamoto, Keiichi Jingu

**Affiliations:** Department of Radiation Oncology, Tohoku University Graduate School of Medicine, 1-1 Seiryo-machi, Aoba-ku, Sendai, Miyagi, 980-8574, Japan; Department of Radiation Oncology, Tohoku University Graduate School of Medicine, 1-1 Seiryo-machi, Aoba-ku, Sendai, Miyagi, 980-8574, Japan; Department of Radiation Oncology, Tohoku University Graduate School of Medicine, 1-1 Seiryo-machi, Aoba-ku, Sendai, Miyagi, 980-8574, Japan; Department of Advanced Biomedical Imaging, University of Yamanashi, 1110 Shimokato, Chuo-city, Yamanashi, 409-3898, Japan; Department of Radiation Oncology, Tohoku University Graduate School of Medicine, 1-1 Seiryo-machi, Aoba-ku, Sendai, Miyagi, 980-8574, Japan; Department of Radiation Oncology, Tohoku University Graduate School of Medicine, 1-1 Seiryo-machi, Aoba-ku, Sendai, Miyagi, 980-8574, Japan; Department of Radiation Oncology, Tohoku University Graduate School of Medicine, 1-1 Seiryo-machi, Aoba-ku, Sendai, Miyagi, 980-8574, Japan; Department of Radiation Oncology, Tohoku University Graduate School of Medicine, 1-1 Seiryo-machi, Aoba-ku, Sendai, Miyagi, 980-8574, Japan; Department of Radiation Oncology, Tohoku University Graduate School of Medicine, 1-1 Seiryo-machi, Aoba-ku, Sendai, Miyagi, 980-8574, Japan; Department of Radiation Oncology, Tohoku University Graduate School of Medicine, 1-1 Seiryo-machi, Aoba-ku, Sendai, Miyagi, 980-8574, Japan; Department of Radiation Oncology, Tohoku University Graduate School of Medicine, 1-1 Seiryo-machi, Aoba-ku, Sendai, Miyagi, 980-8574, Japan

**Keywords:** radiotherapy, deep learning, machine learning, automated planning, dose mimicking, lung cancer

## Abstract

This study aimed to evaluate the clinical validity of a dose-mimicking automated planning for volumetric-modulated arc therapy (VMAT) in patients with stage III non-small cell lung cancer (NSCLC), through direct comparison with a commercial dose volume histogram (DVH)-predicted system. We retrospectively analyzed volumetric-modulated arc therapy plans from 75 patients with stage III NSCLC treated at our institution (60 for training, 15 for testing). The dose-mimicking method was implemented using RatoGuide, and the DVH-predicted method was implemented using RapidPlan. The RatoGuide 3D dose-prediction model was trained on the 60 training cases. For each test case, a predicted dose distribution was generated and converted to a deliverable plan (RGDose) in Eclipse using vendor-provided objective functions. A RapidPlan model trained and generated deliverable plans (RPDose) for the same dataset. The clinical plan dose distribution (CliDose) was the reference. We compared dose distributions and DVH parameters among RGDose, RPDose and CliDose. Mean absolute errors (MAEs) relative to CliDose were 0.83 ± 0.66% (targets) and 2.06 ± 3.14% (organs at risk [OARs]) for RGDose, and 0.88 ± 0.66% (targets) and 2.49 ± 3.63% (OARs) for RPDose. There were no significant differences in OAR DVH parameters between RGDose and CliDose. In contrast, compared to CliDose, RPDose showed a significant reduction in the Esophagus D1cc and a significant increase in the Lungs V5Gy. The dose-mimicking method more faithfully reproduced the original clinical plans than the conventional DVH-predicted system, suggesting that dose-mimicking method can capture complex inter-OAR trade-offs and consistently reflect planner intent.

## INTRODUCTION

High-precision radiotherapy techniques such as intensity-modulated radiation therapy (IMRT) and volumetric-modulated arc therapy (VMAT) enable highly conformal dose delivery to tumors while sparing surrounding organs at risk (OARs) [1, 2]. However, the inverse planning process required for these techniques presents two major clinical challenges: the quality of the treatment plan is subject to inter-planner variability, and the planning process itself is highly time-consuming [3, 4].

To address these issues, artificial intelligence (AI)-based automated planning has been actively developed, broadly categorized into two approaches: dose-volume histogram (DVH)-predicted methods and dose-mimicking methods. The DVH-predicted method optimizes plans to reproduce one-dimensional (1D) DVHs predicted from patient anatomy. This method is widely implemented in the clinic through the commercial system RapidPlan (Varian Medical Systems, Palo Alto, CA). Tol *et al*. evaluated RapidPlan for head-and-neck VMAT and claimed that its plans were comparable or superior to clinical plans, with improved target-dose homogeneity and significantly lower mean doses to swallowing structures [5]. Hirashima *et al*. evaluated RapidPlan models for head and neck, pancreatic and rectal cancer, concluding that the dosimetric indices of their plans were comparable to clinical plans regardless of energies, multileaf collimator (MLC) types and disease sites [6]. However, this method has limitations such as the inherent loss of three-dimensional spatial information, as the DVH is merely a statistical summary of dose distribution rather than a map of voxel-level doses [7, 8]. Recently, the dose-mimicking method has been developed. This method generates plans by optimizing to mimic a predicted three-dimensional (3D) dose distribution, thereby believed to enable more precise plan automation through the preservation of spatial dose information lost in DVHs. Fan *et al*. developed a method to predict 3D dose distribution using deep learning (DL) and then perform voxel-based optimization using the prediction as a goal [9]. Miki *et al*. devised a technique that uses ring structures, created from the predicted dose distribution, as optimization objectives [10]. These reports indicate the potential of dose-mimicking methods to create clinically acceptable plans.

Patients with stage III non-small-cell lung cancer (NSCLC) who are medically or surgically inoperable and with good performance status should be offered concurrent chemotherapy and radiation therapy [11, 12]. High-precision radiotherapy techniques, such as IMRT and VMAT, have become the predominant therapeutic modality for stage III NSCLC, utilized in over 90% of cases [13]. However, automated IMRT or VMAT planning for stage III NSCLC is considered challenging owing to its high anatomical diversity, with significant inter-patient variability in tumor size, location and spatial relationships with multiple OARs of different natures. Underscoring this difficulty, an AI-based planning study by Fogliata *et al*., which evaluated lung and prostate cases, reported that the rate of unmet dose constraints was considerably higher in the lung group (10.1%) than in the prostate group (3.1%) [14]. To the best of our knowledge, no study has yet reported the clinical evaluation of a dose-mimicking method to create deliverable plans for patients with stage III NSCLC. Barragán-Montero *et al*. proposed a deep learning-based 3D dose prediction model for lung IMRT patients, incorporating heterogeneous beam configurations. However, their work focused solely on dose distribution prediction and did not proceed to generate or clinically evaluate deliverable treatment plans [15].

Recently, a prototype DL-based system, RatoGuide (AiRato Inc, Sendai, Japan), was developed. RatoGuide is a treatment planning support software that first predicts a 3D dose distribution via DL and then generates a deliverable plan that reproduces this prediction using vendor-supplied objective functions. The clinical feasibility of RatoGuide has been demonstrated in prostate and gynecologic cancers [16, 17].

The primary purpose of this study was to evaluate the clinical validity of deliverable plans for stage III NSCLC created by the dose-mimicking method using RatoGuide. To benchmark its performance, we compared these plans against those generated by RapidPlan, a widely adopted commercial software that represents the conventional DVH-predicted method.

## MATERIALS AND METHODS

### Patient information

This retrospective study was approved by our institutional review board (2025–1-119). We retrospectively collected treatment planning data from 75 patients with stage III NSCLC who underwent VMAT at our institution between 2019 and 2024. The data were split into a training set of 60 cases and a test set of 15 cases. Patient characteristics are shown in Table 1. All patients underwent CT simulation on a SOMATOM Definition AS+ scanner (Siemens, Munich, Germany). The CT slice thickness was 2 mm, and the pixel size was 1.3 mm.

**Table 1:** Patient characteristics

		Training (*n* = 60)	Test (*n* = 15)	*P*-value
Age		67.8 (21–84)	71.3 (54–83)	
Gender	Male	50	14	
	Female	10	1	
Tumor location	Right	32	7	
	Left	28	8	
PTV Volume [cc]		376.0 (41.7–985.8)	418.1 (48.5–955.3)	
DVH parameters (mean ± SD)	PTV_D95% [%]	96.2 ± 0.8	95.3 ± 0.9	0.0012
	CTV_D98% [%]	97.4 ± 0.8	96.9 ± 0.7	0.0313
	Spinalcord_Dmax [Gy]	33.4 ± 8.9	32.1 ± 4.1	0.1279
	Spinalcord_PRV_Dmax [Gy]	35.9 ± 9.6	34.4 ± 5.2	0.1221
	Esophagus_D1cc [%]	77.7 ± 29.1	78.8 ± 27.1	0.9101
	Esophagus_Dmean [Gy]	15.8 ± 9.5	17.5 ± 9.9	0.9165
	Heart_V63Gy [%]	0.0 ± 0.0	0.0 ± 0.0	0.6227
	Heart_V50Gy [%]	2.3 ± 2.5	1.1 ± 1.5	0.1509
	Heart_Dmean [Gy]	9.2 ± 6.3	6.2 ± 6.1	0.0758
	Lungs_V20Gy [%]	16.7 ± 6.2	18.0 ± 4.4	0.3929
	Lungs_V5Gy [%]	36.0 ± 12.5	42.3 ± 11.9	0.1120
	Lungs_Dmean [Gy]	9.9 ± 3.1	10.8 ± 2.3	0.2083

### Contour information

All structures were delineated by experienced radiation oncologists. The Planning Target Volume (PTV) was defined by adding a 5.0–7.0 mm margin around the Clinical Target Volume (CTV). The OARs delineated were the Lungs, Esophagus, Heart and Spinal cord. A 3.0 mm margin was added to the Spinal cord to create a planning organ at risk volume (PRV), named Spinalcord_PRV.

### Plan information

All treatment plans were prescribed to 60 Gy in 30 fractions. The plans were created using the Eclipse Treatment Planning System (Varian Medical Systems, Palo Alto, CA) with 6 MV photon energy and a two half-arc VMAT technique (0° to 179° or 181° to 0°). The collimator angles were set to 30° for the first arc and 330° for the second arc. The AcurosXB algorithm was used for dose calculation on a TrueBeam STx linear accelerator, with a dose grid size of 2 mm. Dose constraints followed our institutional clinical protocol, as shown in Supplementary Table S1. All plans were normalized such that 50% of the PTV received 100% of the prescribed dose. For the 15 case test set, the dose distribution of the clinical plans in the test dataset was defined as CliDose.

### Study workflow

**Fig. 1:**
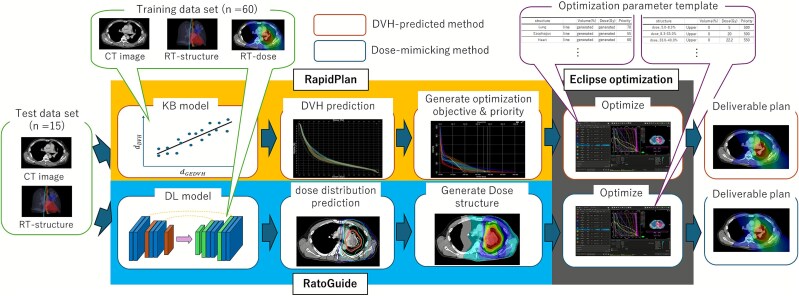
Study workflow. We evaluated two automated planning methods for VMAT of stage III NSCLC: the dose-mimicking method (RatoGuide) and the DVH-predicted method (RapidPlan). First, models were trained using a training dataset of 60 cases. In the dose-mimicking method, the trained DL model predicted 3D dose distributions for 15 test cases. Optimization structures were generated from these predicted doses and imported into the Eclipse TPS. Deliverable plans (RGDose) were then created by performing optimization in Eclipse using vendor-provided parameters. In the DVH-predicted method, a RapidPlan model was created using the same dataset. Deliverable plans (RPDose) were generated by optimizing based on the predicted achievable DVHs in Eclipse. Finally, the two automated plans (RGDose and RPDose) were compared with the manual clinical plans (CliDose) in terms of dose distribution, DVH parameters and 3D similarity.

Figure 1 illustrates the overall workflow of this study, comparing the two automated planning methods evaluated. The dose-mimicking method was implemented using RatoGuide, and the DVH-predicted method was implemented using RapidPlan. For both methods, plan optimization was performed using the Eclipse treatment planning system (TPS), utilizing the same optimization engine. Since RatoGuide’s vendor-supplied parameters are designed specifically for Eclipse, this shared optimization environment eliminates algorithmic discrepancies, ensuring that observed differences primarily reflect the characteristics of the respective AI methods.

### Dose-mimicking method planning

The workflow for training and plan generation using RatoGuide comprised four steps:

Step 1: Training of the DL model. The DL model is based on the Dense Dilated U-Net architecture proposed by Gronberg *et al.* [18]. The model was trained on the training data, which included CT images, RT-Structures (Body, PTV, CTV, Esophagus, Heart, Lungs, Spinal cord), and the corresponding RT-Dose. To standardize the training dataset, which was initially planned by multiple medical physicists, a data cleansing process was performed. This involved replanning 38 of the 60 training cases by a single experienced medical physicist to ensure consistency.

Step 2: Creation of predicted dose distribution. The planning CT images and RT-Structures of the test data were imported into RatoGuide. The seven organ contours (Body, PTV, CTV, Esophagus, Heart, Lungs, Spinal cord) from the imported data were mapped to the corresponding structures in RatoGuide. After inputting the prescription dose (60Gy), the DL model generated a predicted dose distribution, which was then normalized in RatoGuide so that 50% of the PTV received 100% of the prescribed dose.

Step 3: Creation of structures for optimization. The predicted dose distribution is an ideal, non-deliverable distribution that does not account for physical constraints (e.g. MLC speed, dose rate). Therefore, to create a deliverable plan, optimization structures were generated from the predicted dose, as shown in Table 2.

**Table 2:** Structure for optimization

Structure	Description
dose_5.0–8.3%	Regions surrounded by 5.0–8.3% of the predicted dose in the body
dose_8.3–33.0%	Regions surrounded by 8.3–33.0% of the predicted dose in the body
dose_33.0–40.0%	Regions surrounded by 33.0–40.0% of the predicted dose in the body
dose_40.0–60.0%	Regions surrounded by 40.0–60.0% of the predicted dose in the body
dose_60.0–80.0%	Regions surrounded by 60.0–80.0% of the predicted dose in the body
dose_70.0–90.0%	Regions surrounded by 70.0–90.0% of the predicted dose in the body
dose_80.0–95.0%	Regions surrounded by 80.0–95.0% of the predicted dose in the body
dose_103.0–110.0%	Regions surrounded by 103.0–110.0% of the predicted dose in the body
Lungs/Dose5.0–8.3%	Regions surrounded by 5.0–8.3% of the predicted dose within only the Lungs
Lungs/Dose8.3–33.0%	Regions surrounded by 8.3–33.0% of the predicted dose within only the Lungs
PTV-Dose99.0–110.0%	Region of PTV excluding Regions surrounded by 99.0–110.0% of the predicted dose
PTV-CTV	Region of PTV excluding CTV

Step 4: Plan optimization. The optimization structures created in Step 3 were imported into Eclipse. The plan settings in Eclipse were configured to match those of the clinical plans. For beam settings, two half-arcs from 181° to 0° were used for tumors in the right lung, and from 0° to 179° for tumors in the left lung. The optimization was performed based on vendor-provided optimization parameters, shown in Table 3. These parameters are designed to be generalized to a wide range of patients, and the optimization was run only once for each patient without any human intervention. The resulting dose distribution was defined as RGDose.

**Table 3:** Optimization parameters for RatoGuide

Structure	Objective type	Volume [%]	Dose [Gy]	Priority
CTV	Upper	0	59.4	800
	Lower	100	60.6	950
PTV	Upper	0	59.4	800
	Lower	100	59.4	950
PTV-CTV	Upper	0	59.4	800
	Lower	100	59.4	950
PTV-Dose99.0–110.0%	Lower	100	59.4	950
dose_5.0–8.3%	Upper	0	5	500
dose_8.3–33.0%	Upper	0	20	500
dose_33.0–40.0%	Upper	0	22.2	550
dose_40.0–60.0%	Upper	0	34.2	600
dose_60.0–80.0%	Upper	0	46.2	700
dose_70.0–90.0%	Upper	0	52.2	800
dose_80.0–95.0%	Upper	0	58.8	800
dose_103.0–110.0%	Upper	0	61.2	700
Esophagus	Upper	0	60	800
Heart	Upper	0	60	800
Lungs/Dose5.0–8.3%	Upper	0	3	950
Lungs/Dose8.3–33.0%	Upper	0	16.2	800
SpinalCord	Upper	0	35	800
SpinalCord_PRV	Upper	0	38	800

### DVH-predicted method planning

To ensure a fair comparison with dose-mimicking method, RapidPlan was trained and tested using the same 60-case training set and 15-case test set, respectively. The generated prediction model was evaluated using Varian’s Model Analytics tool to identify and exclude outliers. RapidPlan automatically sets optimization objectives based on the predicted achievable DVH. The types of optimization objectives must be predefined; for this study, they were set according to our institution’s template, as shown in Table 4. ‘Line Objectives’ were used for all OARs except the Spinal cord. Since the maximum dose is the critical parameter for the Spinal cord, a serial organ, a max dose objective was set instead of predicting the entire DVH. Plan settings were identical to the clinical plans, and the optimization was run only once for each patient. The resulting dose distribution was defined as RPDose.

**Table 4:** Optimization parameters for RapidPlan

Structure	Objective type	Volume [%]	Dose [Gy]	Priority
CTV	Upper	0	59.4	80
	Lower	100	60.6	100
PTV	upper	0	59.4	80
	lower	100	59.4	100
PTV-CTV	Upper	0	59.4	80
	Lower	100	59.4	100
Lungs	line	generated	generated	generated
Esophagus	line	generated	generated	generated
Heart	line	generated	generated	generated
Spinalcord	upper	0	35	generated
SpinalCord_PRV	upper	0	38	generated
Normal tissue objective		Distance from target = 0.5 cm		100
		start dose = 100%		
		End dose = 40%		
		Fall-off = 0.25 cm		

### Evaluation methods

The clinical validity of the three dose distributions (RGDose, RPDose and CliDose) generated for the 15 patients in the test set was assessed as follows:

1. DVH parameters were compared based on the dose constraints in Supplementary Table S1. Statistical comparisons were performed for two comparisons: RGDose vs. CliDose, and RPDose vs. CliDose.
2. To evaluate the similarity of the automated plans to the clinical plans, the isodose Dice similarity Coefficient (iDSC) was calculated for the 3D dose distributions. The iDSC was calculated using equation (1), as originally introduced by Liu *et al*. [19]:
  (1) \begin{align*} iDSC=\frac{2\left|A\cap B\right|}{\left|A\right|+\left|B\right|} \end{align*}
  where A is the isodose volume from CliDose and B is the isodose volume from the automated plan (RGDose or RPDose). The iDSC was calculated for isodose volumes ranging from 1% to 95% of the prescription dose in 1% increments. The iDSC ranges from 0 to 1, with values closer to 1 indicating higher volumetric similarity.
3. To quantify the local dose deviations between the automated plans (RGDose and RPDose) and CliDose, a voxel-wise 3D dose difference analysis was performed. Following the methodology described by Liu *et al*., the analysis was conducted on all voxels within the Body contour [19]. For each voxel $i$, the dose difference $DD(i)$ between the automated plan (${D}_{Automated}(i)$) and the clinical plan (${D}_{CliDose}(i)$) was calculated using Equation (2):
  (2) \begin{align*} DD(i)={D}_{Automated}(i)-{D}_{CliDose}(i)\ \end{align*}
  The calculated dose difference $DD(i)$ was normalized to the prescription dose (60Gy) and expressed as a percentage (%). For each patient, the Mean Absolute Error (MAE), defined as the average of the absolute differences $\mid DD(i)\mid$, was calculated to assess the overall magnitude of the error. Furthermore, data from all patients were aggregated, and the percentage of ‘passing’ voxels—those with an absolute dose difference $\mid DD(i)\mid$ below a given threshold (ranging from 1% to 20%)—was calculated and plotted as a Dose Difference Pass Rate curve.
4. Since AI-based techniques have been reported to increase plan complexity, the Modulation Complexity Score for VMAT (MCSv) was calculated to quantify the modulation and complexity of the plans [20, 21]. The MCSv, adapted for VMAT from the original score proposed by McNiven *et al*., evaluates plan complexity based on the variability of both aperture area and leaf positions between adjacent control points [22, 23]. The score ranges from 0 to 1, where a lower value indicates a higher degree of modulation and complexity.
5. The time required to generate each plan with RatoGuide and RapidPlan was recorded. To simulate a realistic clinical workflow, the timing for each method started from the moment the patient’s CT was displayed on the TPS and ended when a deliverable, normalized plan was generated. Specifically, the process for RatoGuide included the time for data transfer between the TPS and RatoGuide, the generation of the predicted dose distribution and optimization structures in RatoGuide, and the subsequent optimization, final dose calculation and normalization within the TPS. For RapidPlan, the time included the prediction of achievable DVHs, optimization, final dose calculation and normalization, all performed in the TPS.

Statistical analysis of DVH parameters was performed using a Wilcoxon signed-rank test in JMP Student Edition 18.2.1 (SAS Institute, Cary). A Bonferroni correction was applied to evaluate the significance of differences between plans, setting the statistical significance level at *P* < 0.05/12 ≈ 0.0042. The iDSC was calculated using MATLAB (MathWorks, Inc.), and the MCSv was calculated using RatoGuide.

## RESULTS

### Dose distributions of a representative case

**Fig. 2:**
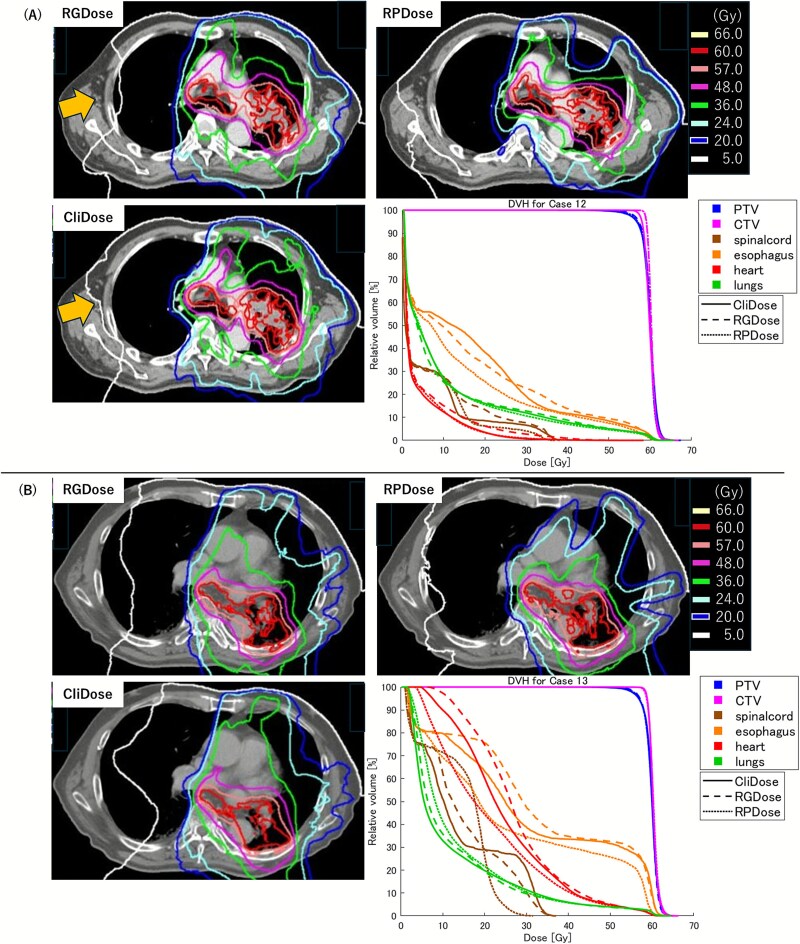
Comparison of dose distribution and DVH in two cases. In case (A), RGDose shows high similarity to CliDose. Arrows indicate reduced low-dose spillage for RGDose. In case (B), RGDose deviates from CliDose and RPDose shows a greater increase in the low-dose lung region.

Figure 2 shows the dose distributions and DVHs of the three plans (RGDose, RPDose and CliDose) for two example cases. Supplementary Table S2 shows the relative errors in DVH parameters between RGDose and CliDose for two example cases. As shown in Fig. 2A, the dose distribution of RGDose was highly similar to CliDose, and the DVHs were visually almost identical. As indicated by the arrows, RGDose reduced the low-dose spillage to the contralateral lung, with the Lungs V5Gy being 3.59% lower than in CliDose. However, in another case shown in Fig. 2B, the dose distributions and DVHs in RGDose were not as similar to CliDose, showing an increase in the low-dose region of the contralateral lung. The Lungs V5Gy for RGDose was 8.34% higher than for CliDose. However, the increase in the low-dose region of the lung was even more pronounced in RPDose, which exhibited the highest Lungs V5Gy among the three treatment plans for this case.

### Comparison of DVH parameters

**Fig. 3:**
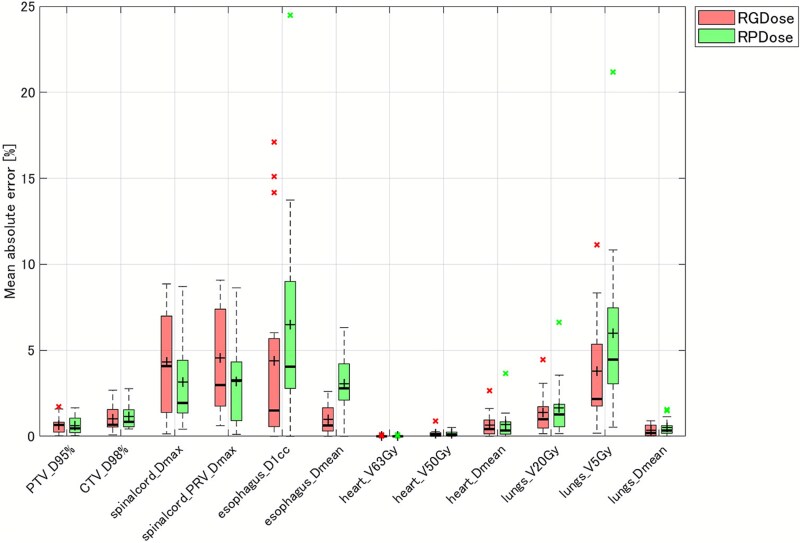
Summary of mean absolute error (MAE) in DVH parameters between RGDose and CliDose, RPDose and CliDose. In each boxplot, the horizontal line represents the median error, and the ‘+’ symbol indicates the mean value. The box indicates the interquartile range (IQR), which contains the middle 50% of the data. The whiskers extend to the furthest data points that lie within 1.5 times the IQR from the box. Outliers beyond this range are plotted as an ×.

Table 5 summarizes the DVH parameters for RGDose, RPDose and CliDose across all patients. Figure 3 shows the mean absolute error (MAE) in DVH parameters between the automated plans and CliDose. The MAE for all target DVH parameters between RGDose and CliDose was 0.83 ± 0.66%, and for OARs, it was 2.06 ± 3.14%. The largest MAE was observed for the Dmax of Spinalcord_PRV at 4.57 ± 3.01%. For RPDose versus CliDose, the MAE for target parameters was 0.88 ± 0.66%, and for OARs, it was 2.49 ± 3.63%. The largest MAE was for the D1cc of the Esophagus at 6.48 ± 6.16%. The MAEs for all DVH parameters, except for the Spinal cord and Spinalcord_PRV, were either comparable or smaller for RGDose than for RPDose.

**Table 5:** Summary of DVH parameters for RGDose, RPDose and CliDose

Structure	DVH metrics	RGDose	RPDose	CliDose	*P*-value	*P*-value
		Mean ± SD	Mean ± SD	Mean ± SD	RGDose vs CliDose	RPDose vs CliDose
PTV	D95% [%]	95.5 ± 0.9	95.2 ± 0.7	95.3 ± 0.9	0.124	0.600
CTV	D98% [%]	97.9 ± 0.5	98.0 ± 0.4	96.9 ± 0.7	<0.001	0.002
Spinalcord	Dmax [Gy]	33.1 ± 7.3	31.2 ± 6.1	32.1 ± 4.1	0.489	0.525
Spinalcord_PRV	Dmax [Gy]	35.7 ± 8.3	34.3 ± 7.3	34.4 ± 5.2	0.525	0.847
Esophagus	D1cc [%]	76.0 ± 29.2	71.9 ± 30.6	78.8 ± 27.1	0.273	<0.001
	Dmean [Gy]	17.4 ± 10.5	14.2 ± 8.7	17.5 ± 9.9	0.903	0.013
Heart	V63Gy [%]	0.0 ± 0.0	0.0 ± 0.0	0.0 ± 0.0	0.500	0.500
	V50Gy [%]	1.3 ± 1.6	1.0 ± 1.4	1.1 ± 1.5	0.010	0.021
	Dmean [Gy]	6.7 ± 6.8	5.7 ± 5.3	6.2 ± 6.1	0.019	0.229
Lungs	V20Gy [%]	18.3 ± 3.9	18.5 ± 4.3	18.0 ± 4.4	0.083	0.135
	V5Gy [%]	40.4 ± 12.8	47.7 ± 15.4	42.3 ± 11.9	0.429	0.002
	Dmean [Gy]	10.8 ± 2.3	11.0 ± 2.4	10.8 ± 2.3	0.590	0.169

*Note*: Statistical significance was determined using a Bonferroni-corrected threshold of *P* < 0.0042 (0.05/12).

Compared to CliDose, RGDose showed a statistically significant improvement in CTV D98% (*P* < 0.001), with no other significant differences in DVH parameters. RPDose also showed a significant improvement in CTV D98% (*P* < 0.001) and a significant decrease in Esophagus D1cc (*P* < 0.001) compared to CliDose. However, it also resulted in a significant increase in Lungs V5Gy (*P* = 0.002).

### Comparison of DVH curves

Supplementary Fig. S1 displays the average DVH curves for the target and OARs for RGDose, RPDose and CliDose. The DVHs of both RGDose and RPDose were generally consistent with that of CliDose. However, RPDose achieved a substantially lower dose to the Esophagus compared to CliDose, while exhibiting a larger low-dose bath to the Lungs (in the region below 20Gy). Regarding target coverage, both AI-generated plans, RGDose and RPDose, were superior to CliDose.

### Comparison of iDSC

**Fig. 4:**
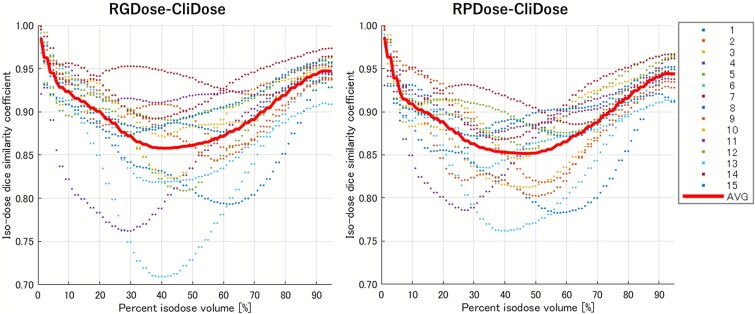
The isodose Dice similarity coefficient (iDSC) between RGDose and CliDose, RPDose and CliDose. iDSC is a metric of volumetric similarity between dose distributions, with values closer to 1 indicating a higher degree of similarity. The red solid line represents the mean iDSC value across all patients.

Figure 4 shows the iDSC values between the automated plans and CliDose for all patients. The red solid line represents the average iDSC. Both RGDose and RPDose exhibited a similar trend, with higher iDSC values in the low- and high-dose regions and lower values in the mid-dose region. Overall, RGDose showed slightly higher iDSC values. The mean iDSC values for both RGDose and RPDose exceeded 0.85 across all dose regions, with RGDose surpassing 0.9 in the low- (0–20%) and high-dose (75–95%) regions. Similarly, the iDSC between the Predicted Dose and RGDose (Supplementary Fig. S2) showed a comparable trend to that between RGDose and CliDose, being high in the low- and high-dose regions, with the mean value reaching a minimum of approximately 0.9 around the 40% isodose volume.

### Voxel-wise 3D dose difference

The results of the 3D voxel-wise dose difference analysis are summarized in Supplementary Fig. S3. Supplementary Fig. S3a illustrates the patient-wise dose difference MAE for all 15 test cases relative to CliDose. The MAE for RGDose was generally comparable to or slightly lower than that for RPDose across most patients. The population average MAE (± standard deviation) was 1.4 ± 0.5% (range, 0.5–2.3%) for RGDose and 1.6 ± 0.7% (range, 0.9–3.6%) for RPDose.

Supplementary Fig. S3b presents the aggregated Dose Difference Pass Rate curves for the entire patient cohort. These curves show the percentage of voxels within the Body contour achieving an absolute dose difference below a given threshold. RGDose consistently demonstrated a higher pass rate than RPDose across all thresholds.

### MCSv and planning time

The mean MCSv values for RGDose, RPDose and CliDose were 0.26 ± 0.02, 0.25 ± 0.03, and 0.26 ± 0.04, respectively. The *P*-values for the comparison between RGDose or RPDose and CliDose were 0.632 and 0.556, respectively. The average planning times for RGDose and RPDose were 20 min and 45 s, and 6 min and 7 s, respectively.

## DISCUSSION

In this study, we conducted the first clinical evaluation of deliverable VMAT plans for stage III NSCLC created using a dose-mimicking method (RatoGuide), assessing them by comparing against deliverable plans from RapidPlan, a system utilizing the conventional DVH-predicted method.

The key findings are twofold. First, RGDose demonstrated slightly higher overall dosimetric fidelity to clinical plans than the RPDose, evidenced by lower MAEs in DVH parameters for both targets and OARs. Second, RGDose successfully reproduced the complex dosimetric trade-offs of the original clinical plans, showing no significant differences in OAR parameters except for improved CTV D98%. In contrast, RPDose selected a different trade-off, achieving a significant reduction in Esophagus D1cc at the cost of a significant increase in Lungs V5Gy. These results suggest that the dose-mimicking method, by learning from holistic 3D spatial information, has a superior capability to capture and replicate the nuanced, expert-driven clinical judgments embedded in treatment plans compared to the DVH-predicted method. The underlying reason for these key findings is likely attributable to the fundamental differences in feature representation and prediction targets. RapidPlan derives achievable 1D DVHs for each OAR from hand-crafted geometric and dosimetric features (e.g. Geometry-Based Expected Dose, OAR-target overlap) [14]. While effective for individual OARs, this approach may struggle to learn high-dimensional information spanning multiple organs, such as complex inter-OAR relationships or implicit trade-offs. In contrast, RatoGuide utilizes a convolutional neural network (CNN) to automatically extract features from the entire 3D anatomical information and directly predict the 3D dose distribution. This enables the learning of higher-dimensional information, such as the spatial relationships and overlaps between OARs and their impact on the target dose. By predicting spatial dose information lost in 1D DVHs, it can learn implicit multi-OAR trade-offs and more faithfully reproduce the original planning intent.

RGDose achieved high dosimetric accuracy relative to CliDose, with MAEs of 0.83 ± 0.66% for target parameters and 2.06 ± 3.14% for OARs. Furthermore, the MAE for all DVH parameters was below 5%. Kadoya *et al*. evaluated deliverable plans created with RatoGuide for prostate IMRT and reported MAEs of 1.32 ± 1.35% for target parameters and 2.08 ± 2.79% for OARs, with all MAEs below 6% [16]. Our results are consistent with their findings, indicating that the dose-mimicking method can generate plans for stage III NSCLC with accuracy comparable to other anatomical sites. Furthermore, RGDose achieved a significantly higher CTV D98% and a lower (though non-significant) Lungs V5Gy compared to CliDose. We attribute this improvement to the rigorous data cleansing of our training set, where suboptimal clinical plans were replanned. This aligns with Fjellanger *et al*., who demonstrated that training models on optimized library plans improves dosimetric outcomes, confirming that enhancing training-data quality directly contributes to superior automated plan quality [24].

Regarding 3D similarity, the mean iDSC values for both RGDose and RPDose exceeded 0.85 across all dose regions, with RGDose surpassing 0.9 in the low- (0–20%) and high-dose (75–95%) regions. This contrasts with a previous breast cancer VMAT study that reported the lowest similarity (<0.9) in the low-dose region [25]. Although a direct comparison is difficult because their predicted dose was non-deliverable, this difference suggests that the AI model can learn the distinct dose distribution characteristics associated with different treatment sites. For stage III NSCLC, reducing the low-dose lung volume is crucial due to its strong correlation with pneumonitis [26, 27]. Therefore, the high accuracy achieved in the low-dose region likely reflects the model’s capability to prioritize this critical requirement specific to NSCLC.

Our 3D similarity metrics revealed two additional points. First, while RGDose demonstrated superior fidelity to CliDose across iDSC (Fig. 4) and the 3D dose difference analysis (Supplementary Fig. S3)—showing slightly higher mean iDSC, lower dose difference MAE (Supplementary Fig. S3a), and consistently higher pass rates (Supplementary Fig. S3b)—the margin of superiority over RPDose was modest. Second, the fidelity scores for RGDose exhibited a larger variance compared to RPDose, an observation apparent in the iDSC plot (Fig. 4). We hypothesize that this modest performance gap stems from the inherent limitations of the conversion process (Steps 3 and 4). The prediction is translated into optimization objectives using ‘Dose Structures’ defined by relatively wide dose ranges, which necessarily approximates the granular 3D prediction. This hypothesis is strongly supported by the comparison between the Predicted Dose and RGDose (Supplementary Fig. S2), where iDSC dipped to approximately 0.9 in the mid-dose region, indicating deviation during optimization. This process likely acts as a ‘bottleneck’ that levels the performance of both methods. While narrower dose ranges could theoretically improve fidelity, preliminary tests showed they often cause convergence failures. Addressing this trade-off is a subject for future investigation. The larger variance in RGDose’s iDSC is likely due to the sensitivity of DL models to ‘edge cases’ and the relatively small training set (*n* = 60), suggesting that increasing data diversity could improve robustness.

Complexity, measured by MCSv, showed no significant difference between automated plans and CliDose, indicating both are clinically deliverable without undue complexity. In terms of efficiency, RGDose and RPDose required 20 min 45 s and 6 min 7 s, respectively. Tohoyama *et al*. reported an average clinical IMRT planning time of 165.6 min, suggesting that AI software can significantly improve planning efficiency [4]. Moreover, RapidPlan can generate a plan in less than one-third of the time required by RatoGuide, which may allow planners to use the saved time for further re-optimization and quality improvement. However, the observed planning time difference reflects not only inherent computational speed but also significant disparities in software implementation; RapidPlan is fully integrated into the TPS, whereas RatoGuide requires time-consuming data transfer as standalone software. Future investigations should aim for a fairer comparison under integrated conditions.

Recently, numerous studies have explored predicting machine parameters (e.g. MLC apertures) directly [28, 29]. Heilemann *et al*. developed a method to generate deliverable VMAT plans by predicting MLC motion sequences and monitor units directly from a predicted dose distribution. Integrating such a machine parameter prediction system into our dose distribution prediction workflow represents a promising future direction to further enhance planning efficiency [30].

This study has two main limitations. First, this study evaluated a single irradiation technique (two half-arc VMAT); other irradiation techniques, such as full-arc VMAT, result in significantly different dose-shape characteristics and would likely require a separate model. Second, the single-institution dataset reflects local planning policies and priorities, which may limit generalizability. Transfer learning offers a practical adaptation pathway, enabling efficient fine-tuning with modest site-specific data to account for institutional differences in constraints and optimization practice [31]. Future work should assess cross-institutional performance and evaluate adapted models under varied beam arrangements.

This study evaluated deliverable VMAT plans for stage III NSCLC created using a dose-mimicking method (RatoGuide), assessing them by comparing against deliverable plans from RapidPlan, a system utilizing the conventional DVH-predicted method. While both methods generated plans comparable to clinical plans, the dose-mimicking method demonstrated superior fidelity to the clinical standard; these results highlight its advantage for handling anatomically complex pathologies like stage III NSCLC, providing valuable insight for the successful clinical implementation of automated planning systems.

## Supplementary Material

Supplementary_Figure_S1_rrag001

Supplementary_Figure_S2_rrag001

Supplementary_Figure_S3_rrag001

Supplementary_information_rrag001

## Presentation at a conference

The content of this manuscript is scheduled to be presented at the 53rd Autumn Scientific Congress of the Japanese Society of Radiological Technology, Sapporo, Japan, October 17–19, 2025.

## Clinical trial registration

Not applicable. This is a retrospective study.
