# Supplementary figures and images for: Evaluation of deliverable dose-mimicking automated volumetric arc radiation therapy planning for stage III non-small cell lung cancer patients: comparison with a commercial DVH-predicted automated planning system
Source: J Radiat Res. 2026 Feb 5;67(2):248–58. doi: 10.1093/jrr/rrag001 (PMC13019132; doi:10.1093/jrr/rrag001)

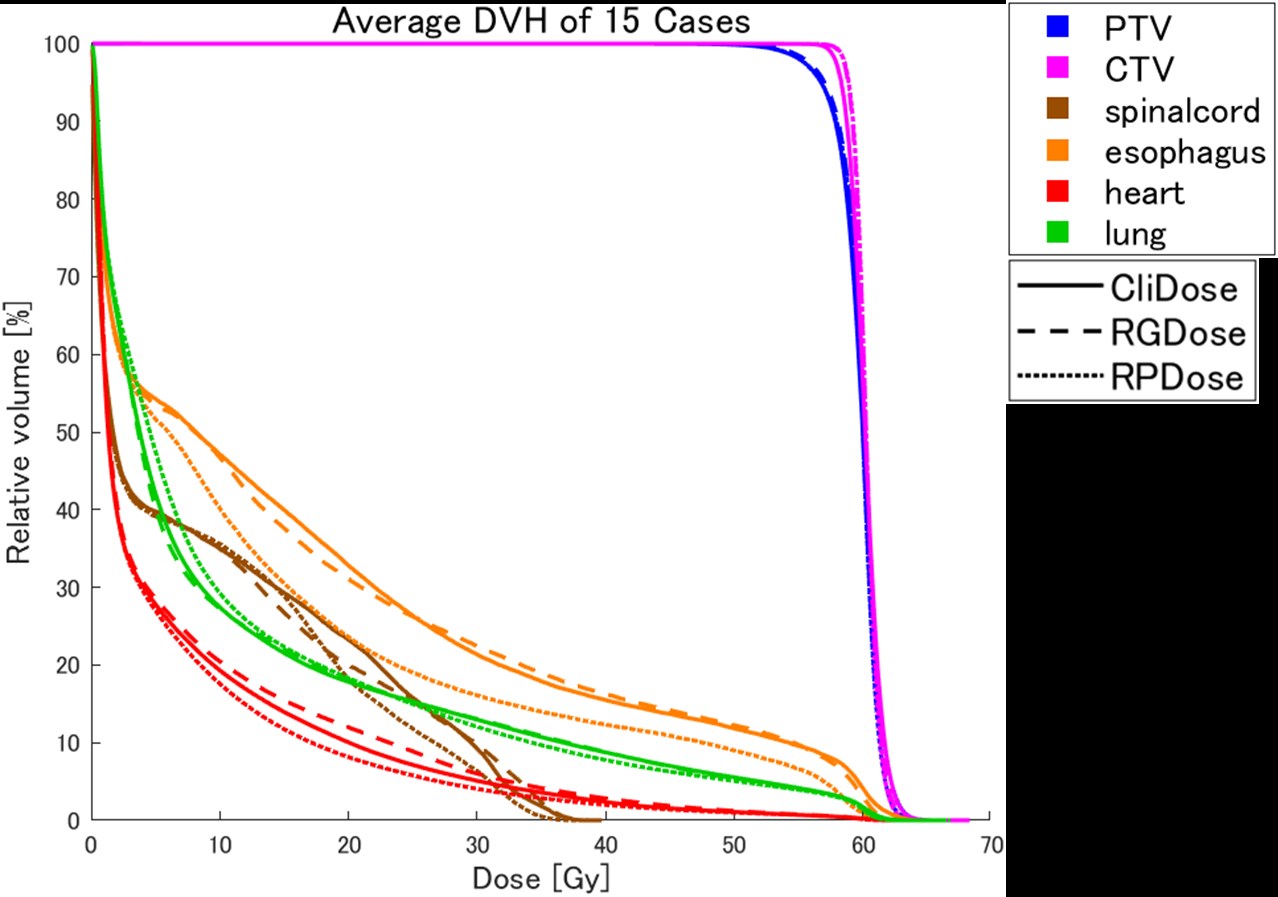

Supplement: Supplementary_Figure_S1_rrag001 [file supplementary_figure_s1_rrag001.jpeg]

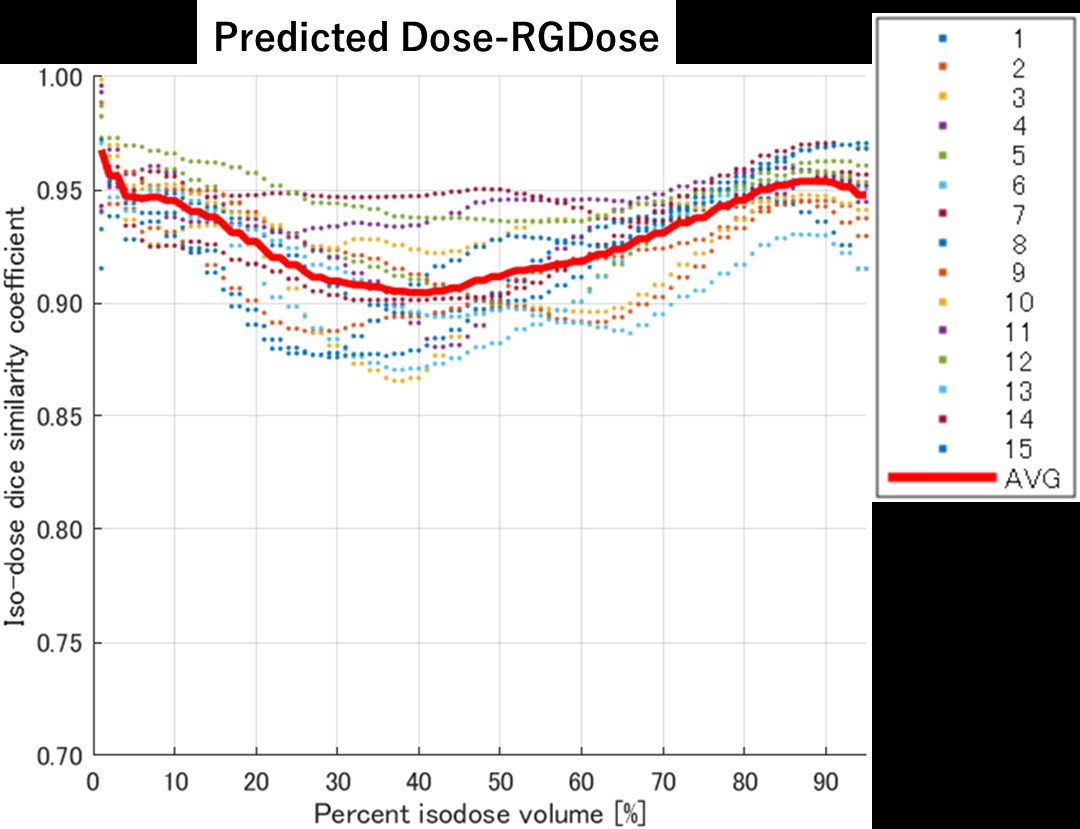

Supplement: Supplementary_Figure_S2_rrag001 [file supplementary_figure_s2_rrag001.jpeg]

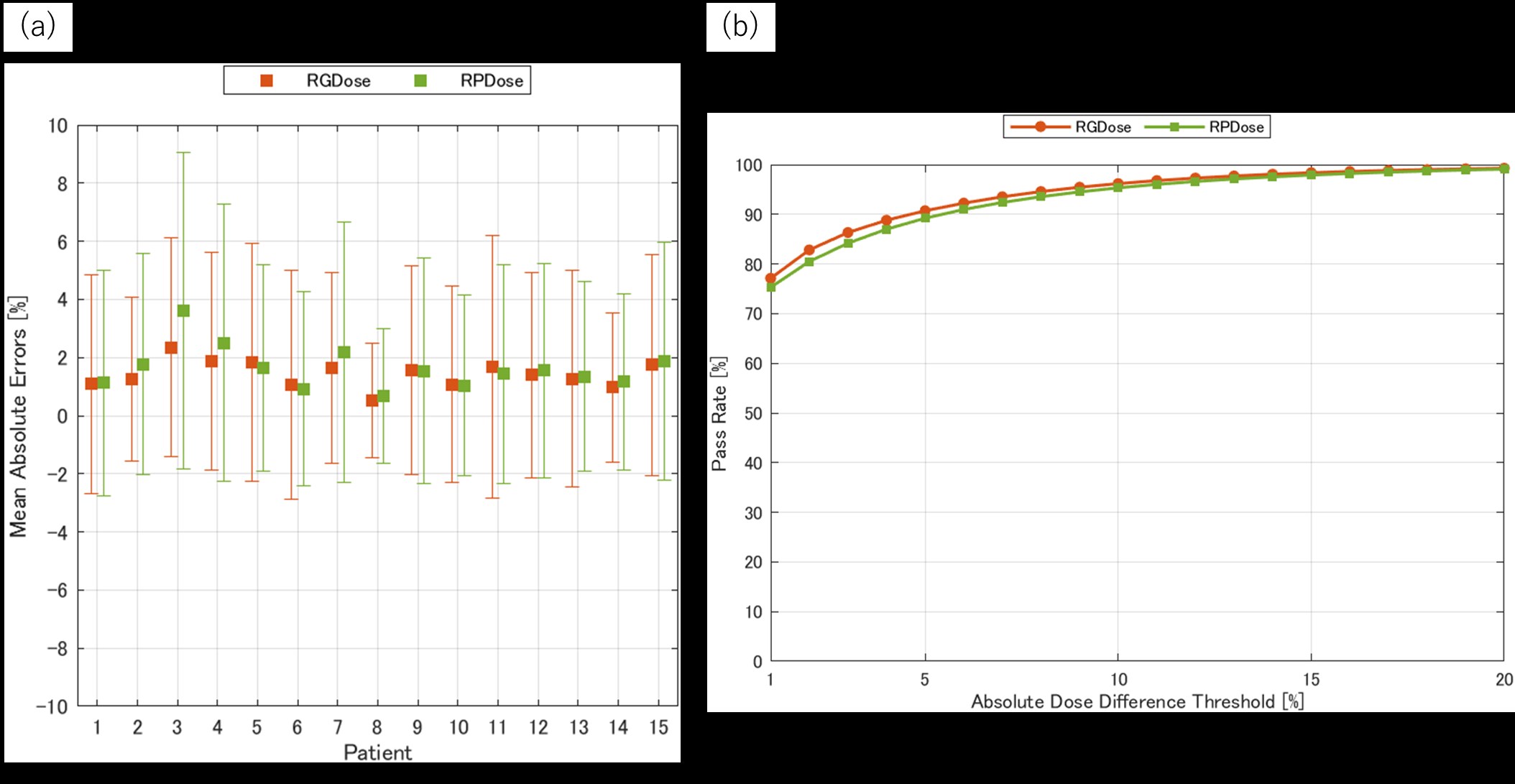

Supplement: Supplementary_Figure_S3_rrag001 [file supplementary_figure_s3_rrag001.jpeg]
