# Supplementary material for: Evaluation of deliverable dose-mimicking automated volumetric arc radiation therapy planning for stage III non-small cell lung cancer patients: comparison with a commercial DVH-predicted automated planning system
Source: J Radiat Res. 2026 Feb 5;67(2):248–58. doi: 10.1093/jrr/rrag001 (PMC13019132; doi:10.1093/jrr/rrag001)
Supplement: Supplementary_information_rrag001 [file supplementary_information_rrag001.docx]

**Supplementary information**

**Evaluation of deliverable dose-mimicking automated volumetric arc radiation therapy planning for stage Ⅲ non-small cell lung cancer patients: comparison with a commercial DVH-predicted automated planning system**

Takeru Nakajima(1), Noriyuki Kadoya(1), Ryota Tozuka(1) (2), Masaki Kondo(1), Shohei Tanaka(1), Kazuhiro Arai(1), Yoshiyuki Katsuta(1), Taichi Hoshino(1), Takaya Yamamoto(1), Keiichi Jingu(1)

(1) Department of Radiation Oncology, Tohoku University Graduate School of Medicine, Sendai, Japan

(2) Department of Advanced Biomedical Imaging, University of Yamanashi, Chuo, Japan

Supplementary Figure S1: **The average DVH curves of OAR and Target in RGDose, RPDose and CliDose for 15 patients.**

Supplementary Figure S2: **The isodose Dice similarity coefficient (iDSC) between the predicted dose distribution generated by RatoGuide (Predicted Dose) and the optimized deliverable plan (RGDose).**

iDSC is a metric of volumetric similarity between dose distributions, with values closer to 1 indicating a higher degree of similarity. The red solid line represents the mean iDSC value across all patients.

Supplementary Figure S3: **Voxel-wise 3D dose difference analysis between automated plans (RGDose and RPDose) and clinical plans (CliDose).**

(a) Patient-wise dose difference Mean Absolute Error (MAE) relative to CliDose for the 15 test cases, calculated for all voxels within the body contour. (b) Aggregated Dose Difference Pass Rate curves for the entire patient cohort. The curves show the percentage of voxels achieving an absolute dose difference below a given threshold.

Supplementary Table S1: **Dose constraints in this study**

| Structure | DVH metric | Optimal | Tolerable |
| --- | --- | --- | --- |
| PTV | D95% [%] | ≧95 | ≧93 |
| CTV | D98% [%] | ≧95 |  |
| Spinalcord | Dmax [Gy] | ≦46 |  |
| Spinalcord_PRV | Dmax [Gy] | ≦48 |  |
| Esophagus | D1cc [%] | ≦105 | ≦110 |
|  | Dmean [Gy] | ≦34 |  |
| Heart | V63Gy [%] | ≦1 |  |
|  | V50Gy [%] | ≦15 | ≦20 |
|  | Dmean [Gy] | ≦20 | ≦25 |
| Lungs | V20Gy [%] | ≦35 | ≦37 |
|  | V5Gy [%] | ≦60 | ≦65 |
|  | Dmean [Gy] | ≦20 | ≦22 |

Supplementary Table S2: **DVH parameter errors between RGDose and CliDose for representative cases (Case A, Case B)**

| structure | DVH metrics | Case A | Case B |
| --- | --- | --- | --- |
| PTV | D95% [%] | 0.49 | 0.37 |
| CTV | D98% [%] | 2.23 | -0.08 |
| Spinalcord | Dmax [%] | 0.79 | 0.35 |
| Spinalcord_PRV | Dmax [%] | 0.62 | 1.64 |
| Esophagus | D1cc [%] | -0.95 | -0.05 |
|  | Dmean [%] | -0.28 | 2.48 |
| Heart | V63Gy [%] | 0 | 0 |
|  | V50Gy [%] | 0.05 | 0.26 |
|  | Dmean [%] | 0.99 | 2.66 |
| Lungs | V20Gy [%] | 0.52 | -0.15 |
|  | V5Gy [%] | -3.59 | 8.34 |
|  | Dmean [%] | -0.01 | 0.37 |
